# The effect of crystal structure on the electromechanical properties of piezoelectric Nylon-11 nanowires†
†Supporting data for this paper is available at the DSpace@Cambridge data repository (https://doi.org/10.17863/CAM.22784).
‡
‡Electronic supplementary information (ESI) available: Experimental procedures, details of characterization results. See DOI: 10.1039/c8cc02530d


**DOI:** 10.1039/c8cc02530d

**Published:** 2018-06-01

**Authors:** Yeon Sik Choi, Sung Kyun Kim, Findlay Williams, Yonatan Calahorra, James A. Elliott, Sohini Kar-Narayan

**Affiliations:** a Department of Materials Science and Metallurgy , University of Cambridge , 27 Charles Babbage Road , Cambridge CB3 0FS , UK . Email: sk568@cam.ac.uk

## Abstract

Crystal structure is crucial in determining the properties of piezoelectric polymers, particularly at the nanoscale where precise control of the crystalline phase is possible. Here, we investigate the electromechanical properties of three distinct crystalline phases of Nylon-11 nanowires using advanced scanning probe microscopy techniques. Stiff α-phase nanowires exhibited a low piezoelectric response, while relatively soft δ′-phase nanowires displayed an enhanced piezoelectric response.

## Abstract 2

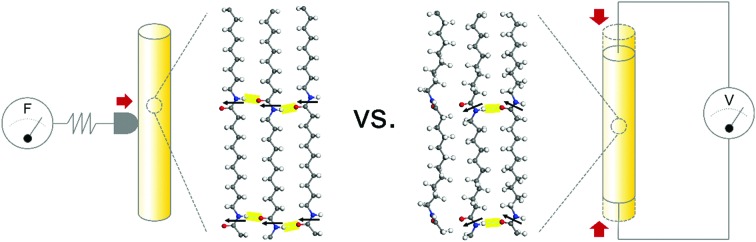
Mechanical and piezoelectric properties of Nylon-11 nanowires strongly depend on their crystal structure, which can be precisely controlled by template-assisted growth methods.

## 

Polymorphism, defined as the existence of several crystalline structures in a material, is of particular interest in piezoelectric polymers as functional properties may vary dramatically across different crystal phases. As a result, the relationship between the crystalline phase and properties of piezoelectric polymers, such as polyvinylidene fluoride (PVDF) and odd-numbered Nylon, has been studied extensively.^1^–^3^ However, the majority of research to date has been conducted on films or on bulk materials.^4^–^6^ In the case of one-dimensional nanowire structures, even though they have many attractive advantages such as a preferential molecular orientation and large surface area, only a limited number of phases and their properties have been realised and studied.^7^–^9^ Therefore, in-depth crystallographic studies of piezoelectric polymer nanowires and their corresponding electromechanical characteristics are crucial for expanding our knowledge of relevant structure–property relationships, as well as widening the range of possible applications, for example as energy harvesters, sensors or actuators.

Here we report the first microscopic mechanical and electrical characterization of Nylon-11 nanowires fabricated in distinct crystalline phases with precise control. Three different nanowire phases were prepared *via* the fine-tuning of a template-assisted nanoconfinement method. The mechanical and electrical properties of these nanowires were extensively explored using scanning probe microscopy techniques, namely quantitative nanomechanical mapping (QNM) and piezoresponse force microscopy (PFM), revealing differences in nanoscale properties across the different nanowires, and when compared to films with the same crystal structure.

The Nylon-11 crystalline phases of particular interest here are the α-phase and δ′-phase (Fig. S1, ESI‡). To realise nanowires of Nylon-11 with different crystalline phases, we modified the conventional template-wetting method which typically involves the infiltration of a polymer solution within nanoporous templates, followed by evaporation of the solvent leading to nanowire formation within the template pores (Fig. 1a and Fig. S2, ESI‡). We note that the speed of crystallization crucially influences the resulting Nylon-11 crystal structure.^10^–^14^ Thus in order to synthesize thermodynamically stable α-phase nanowires, we created slow crystallization conditions within the nanopores. This was achieved by infiltrating Nylon-11 solution in formic acid within anodized aluminium oxide (AAO) templates that were held in a loosely sealed Petri-dish with gentle heating (∼40 °C). The solvent vapour in this roughly sealed condition effectively suppressed the evaporation of remnant solvent. Simultaneously, the gentle heating generated sufficient chain mobility to form an ordered crystal structure within the nanopores. In contrast, fast crystallization conditions led to the formation of metastable δ′-phase nanowires. This was achieved by flowing air over the surface of the AAO template to increase the solvent evaporation rate thus causing extremely fast crystallisation of Nylon-11 within the template pores.^7^ Finally, to serve as a reference sample, we fabricated Nylon-11 nanowires by “conventional” template-wetting.^8^ In this case, the crystallization process occurred at a moderate speed in an open Petri-dish without heating or assisted gas flow.

**Fig. 1:**
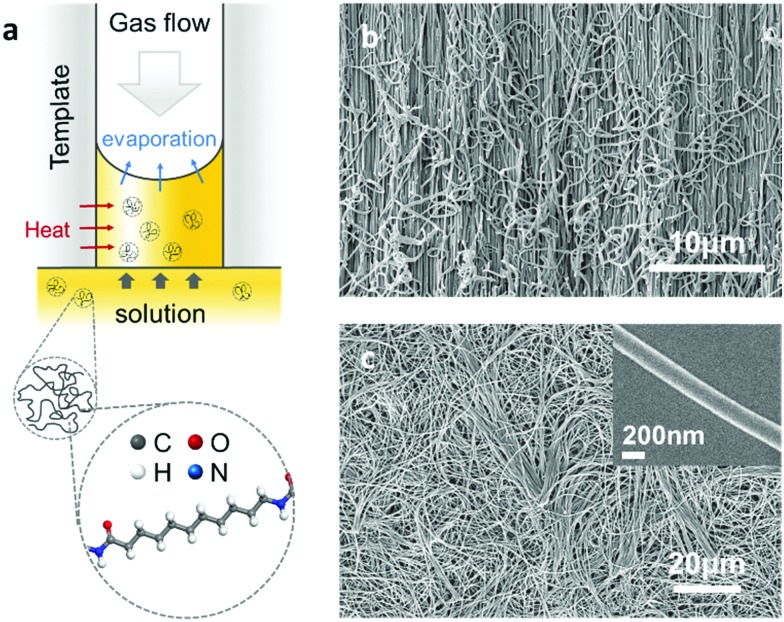
(a) Schematic of template-wetting method and processing parameters with a simulated molecular structure of Nylon-11. (b and c) SEM images of template cross-section and template-freed nanowires respectively. Inset shows a single Nylon-11 nanowire strand.

The morphology of the resulting nanowires was investigated using high-resolution scanning electron microscopy (SEM). The SEM images show the nanowires grown within the nanoporous AAO template upon cleaving (Fig. 1b), and upon dissolution of the template by phosphoric acid (Fig. 1c). The morphology of a single nanowire strand is shown in the inset of Fig. 1c; the uniform width of about 200 nm was in agreement with the nominal size of the template pores.

Three different crystal structures of Nylon-11 nanowires were identified by X-ray diffraction (XRD) analysis (Fig. 2a). α-Phase nanowires (orange) were characterized by three reflections located at 2*θ* = 7.8°, 20°, and 24.2°. These peak positions correspond to the (001), (200), and (210/010) planes of the reported triclinic α-phase Nylon-11 film respectively (Fig. S3, ESI‡).^15^ This means that the slow crystallization and low driving force induced by the heating process generated nanowires with the most thermodynamically stable crystal structure. In the case of δ′-phase nanowires (blue), the diffractograms exhibited two diffraction peaks at 2*θ* = 6.2° and 21.6°, corresponding to the (001) and (*hk*0) planes of pseudo-hexagonal δ′-phase Nylon-11 respectively.^7^,^15^ This suggests that assisted gas-flow during template wetting significantly increased the rate of crystallization, resulting in a metastable crystal structure. Lastly, relatively weak reflections at 2*θ* = 6.2°, 21.6°, and 22.8° were achieved in nanowires grown by conventional template-wetting process (black). Although the corresponding crystal structure has not been previously reported in the bulk, these diffraction peaks agree with the results in our earlier work on Nylon-11 nanowires.^8^

We further verified the direction of molecular orientation of each sample by XRD investigation of nanowires *within* the AAO template (Fig. 2b and Fig. S4, ESI‡). In Bragg–Brentano geometry, only lattice planes with scattering vectors (*q*) normal to the sample surface produce diffraction peaks. The discrepancy in the diffraction patterns between the vertically aligned nanowires and randomly positioned nanowires therefore reveals the preferential chain orientation in the nanowires. When we conducted the XRD measurement on the nanowires in the AAO template, α-phase nanowires (orange) only showed one distinct sharp peak at 2*θ* = 20.0° corresponding to the (001) plane, indicating that the chain axis was perpendicular to the nanowire growth direction.^16^–^18^ In the case of δ′-phase nanowires (blue), a single diffraction peak was observed at 2*θ* = 21.5°. The absence of an (001) peak indicates that the δ′-phase nanowires also had a chain direction perpendicular to the template wall. In the case of Nylon-11 nanowires grown by conventional template-wetting, one recognizable diffraction peak was observed at 2*θ* = 22.8° as compared to the XRD pattern of the corresponding template-freed nanowires. Although there is insufficient data regarding the peak position of conventionally grown nanowires, preferential chain orientation in these nanowires could still be inferred from the differences in the diffractograms. Based on the XRD peak information, we calculated the crystal size using the Scherrer equation (Fig. S5, ESI‡). α-Phase nanowires were found to have the largest crystal size of 21 ± 2.1 nm, and relatively smaller crystal sizes of 11 ± 1.3 nm and 10 ± 1.1 nm were observed from δ′-phase and conventionally grown nanowires, respectively. The variation in crystal size across the different nanowires was as expected based on the respective processing conditions. The large crystal size of α-phase nanowire could be attributed to the slow crystallization speed with sufficient chain mobility from gentle heating. In contrast, fast crystallization inhibited the growth of crystals, resulting in relatively small crystal size in nanowires grown by the other two methods.

**Fig. 2:**
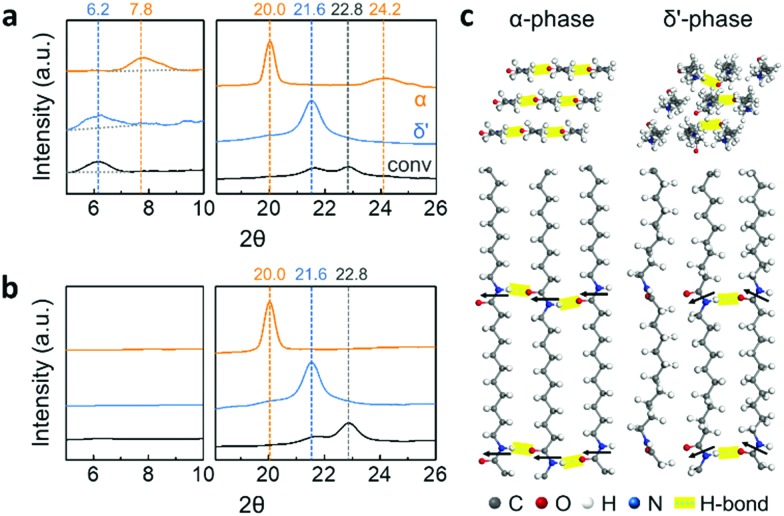
XRD patterns of Nylon-11 nanowires (a) without and (b) with a nanoporous AAO template: α-phase nanowires (orange); δ′-phase nanowires (blue); and nanowires by conventional method (black). (c) 3D rendered images of α and δ′-phase crystal structures: top and side views. Black arrows indicate the direction of amide dipoles.

Based on XRD results, three dimensional molecular images of α and δ′-phase Nylon-11 have been rendered using Materials Studio 8.0 (Fig. 2c).^19^ In the case of the stable α-phase, fully extended chains are packed, and amide chains point to the same direction, forming hydrogen bonding sheets. In contrast, the metastable δ′-phase shows disordered molecular structures with random orientation of hydrogen bonding.^20^

QNM was used to explore the mechanical properties of the different crystal structures in a single nanowire.^21^,^22^ QNM, using an atomic force microscope (AFM) operated in tapping-mode, can be used to simultaneously map sample topography and elastic modulus with nanoscale resolution *via* the analysis of force–distance curves at every pixel of the scanned area.^23^,^24^ The QNM calibration procedure has been separately described in detail (Fig. S6, ESI‡). Fig. 3 shows the height changes across the α-phase nanowire strand mounted on a silicon substrate, while height-correlated trends were presented in the corresponding Derjaguin–Muller–Toporov (DMT) modulus mapping.^25^ However, since the nanowire modulus is not a function of its thickness, the topography-related modulus was assumed to have resulted from tip–nanowire interaction.^21^,^22^ Therefore, a reliable DMT modulus could be achieved by recording the measurement at the top of the nanowire.

**Fig. 3:**
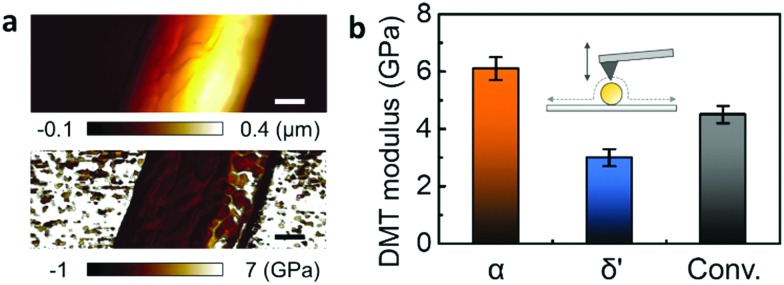
QNM characteristics of various nanowire phases. (a) Height (above) and DMT modulus (below) mapping of α-phase nanowire. (b) Average DMT modulus of α-phase (orange), δ′-phase (blue) and conventional (black) nanowires.

The average DMT modulus of nanowires of different Nylon-11 crystal structures is plotted (Fig. 3b and Fig. S7, ESI‡). The α-phase nanowire (orange) showed the highest modulus (6.1 GPa), which is double that of δ′-phase nanowire (blue, 3 GPa). Nanowires prepared through the conventional template-wetting method (black) exhibited an intermediate modulus of 4.5 GPa. The difference in respective moduli of the different nanowire samples can be explained by the corresponding crystal structures. Firstly, the stiffness of the α-phase nanowires can be attributed to the hydrogen bonding in α-phase Nylon-11 and the relatively large crystal size. Since the polymer chains have sufficient time to align and stack up during crystallization, α-phase Nylon-11 typically contains strong hydrogen bonding with higher crystallinity, resulting in good mechanical properties.^3^,^26^,^27^ In contrast, the δ′-phase exhibits suppressed hydrogen bonding and lower crystallinity due to fast-crystallization,^3^,^26^ thus resulting in much lower DMT modulus. In the case of nanowires prepared *via* a conventional template-wetting method, the only difference between the α-phase growth method and conventional template-wetting method is the growth temperature, which is related to the driving force for chain alignment. The latter therefore has sufficient time to crystallize but does not have enough energy to produce larger crystals with more ordered structure. Therefore, the reduced hydrogen bonding compared with α-phase nanowires explains the comparatively lower modulus of conventionally grown nanowires.

It should be noted that the DMT model is not generally suitable for the analysis of cylinder indentation.^25^ To address this issue, we performed finite-element simulations of nano-indentation on nanowires and thin films (Fig. 4). The indentation force due to the AFM tip on both the nanowires and the films was characterized by changing the sample deformation depth (*d*) and sample thickness. It can be seen that, as the film becomes thinner, the required force to deform the film increases. In the case of the nanowire, however, this effect is reduced and is almost constant due to the suppressed circumferential clamping of a nanowire.^21^,^22^ In addition, the film requires a much higher force for a given deformation than a nanowire, which is more pronounced for larger deformation (6 nm, circle). Thus, nanowires are inherently softer than films of the same intrinsic mechanical properties, and are less affected by the substrate upon reduction of thickness. This suggests that the DMT model actually underestimates the Young's modulus of nanowire samples. In spite of this, in our QNM experiments, we found that α-phase nanowires exhibited much higher DMT modulus (6.1 GPa) than that of α-phase film (2.5 GPa) (Fig. S8, ESI‡), even when the deformation range was similar to that of the simulation (Fig. S9, ESI‡). Our results indicate that due to the large amount of hydrogen bonding and well-aligned crystal structure in the nanowire, α-phase nanowires show much higher mechanical strength than the corresponding film.

**Fig. 4:**
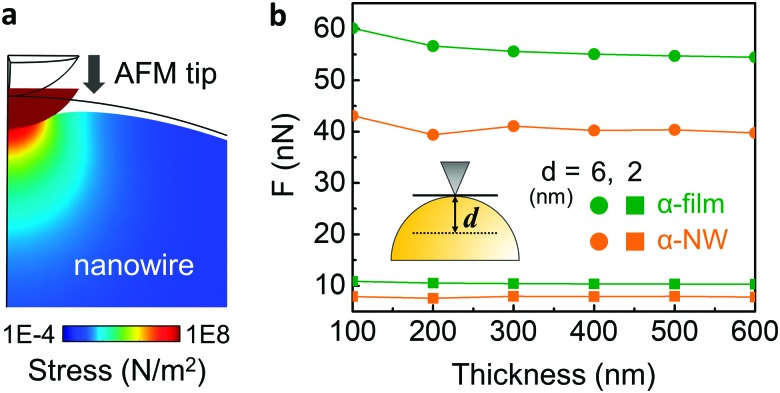
Numerical simulation results of nano-indentation: (a) cross-sectional view of a 6 nm indentation of a nanowire using a 25 nm AFM tip. (b) Indentation force change as a function of thickness of the film (green) and the nanowire (orange). The simulation was conducted using deformation depth (*d*) of 6 nm (circle) and 2 nm (square).

In addition to the mechanical properties, the piezoelectric response of the three different nanowire phases was characterized by PFM (Fig. 5).^22^,^28^ Note that in the case of nanoconfined Nylon-11 nanowires, the additional poling process for PFM analysis is not necessary as self-polarization occurs through preferential molecular orientation.^7^,^8^ With the polarisation direction being parallel to the nanopore axis, the PFM measurement was performed at the top surface of the nanowires filled AAO template.^17^,^29^ As shown in Fig. 5a, the top surface of AAO template comprised the tips of the δ′-phase nanowires. An oscillating piezo-response was observed upon the application of AC bias between the tip and sample (Fig. 5a and Fig. S10, ESI‡). The average deflection amplitude resulting from the piezoelectric response changed as a function of AC bias, as plotted in Fig. 5b. The piezo-response amplitude of δ′-phase nanowires was proportional to the AC bias with a slope of 0.264 mV V^–1^, which suggests that δ′-phase nanowire is piezoelectric (piezoelectric coefficient, *d*_33_ = 3.22 pm V^–1^, see Fig. S11, ESI‡ for details of this estimate). In contrast, α-phase and conventionally grown nanowires do not display significant piezoelectric response (Fig. S12 and S13, ESI‡). It is known that, among various phases of Nylon-11, δ′ is the crystal structure which exhibits the highest piezoelectric response.^3^,^24^,^28^–^31^ However, in the δ′-phase Nylon-11 films, piezoelectric response can be observed only after drawing and/or electric. The δ′-phase nanowires, however, showed a distinct piezoelectric response even without high-voltage poling as a result of the nanoconfinement effect which gives rise to self-poling in these nanowires during growth.^7^,^9^,^34^ On the contrary, in the case of the α-phase nanowires, even though they exhibited a preferential crystal orientation along the nanowire axis, no significant piezo-response was observed. This suggests that strong hydrogen bonding in the α-phase nanowires not only enhanced the nanomechanical properties, but also suppressed the response to an external electric field. This observation is in agreement with reported results from Nylon-11 film of similar crystalline structure.^3^,^26^,^31^–^33^

**Fig. 5:**
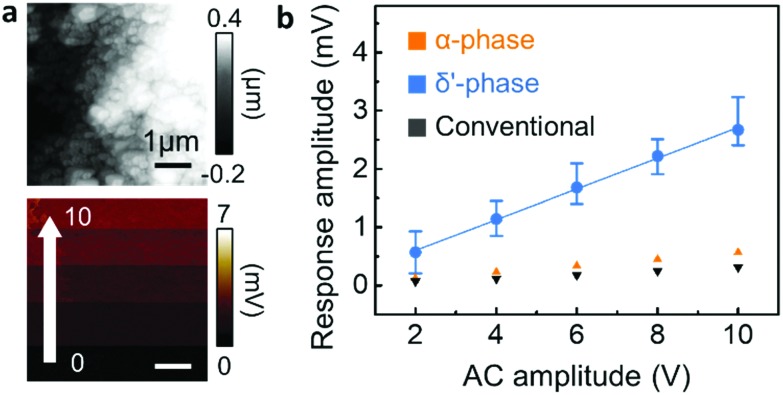
PFM characteristics of various nanowire phases. (a) Height (above) and amplitude (below) mapping of the template's top surface filled with δ′-phase nanowires. (b) Piezoelectric response amplitude of α-phase (orange), δ′-phase (blue) and conventional (black) nanowires as a function of AC amplitude.

In summary, three different nanowire phases of Nylon-11 were prepared *via* the fine-tuning of a template-assisted nanoconfinement method. The mechanical and electrical properties of these nanowires have been explored by QNM and PFM analysis respectively. The α-phase nanowires showed the highest Young's modulus but did not exhibit significant piezoelectric behaviour. In contrast, a distinct piezoelectric response was achieved from the relatively soft δ′-phase nanowires. A similar trend in the variation of properties across the different crystal phases was observed in Nylon-11 films. However, compared to the properties of films with the same crystal structure, the modulus of α-phase nanowires and the piezoelectric response of δ′-phase nanowires was larger. This suggests that the nanoconfinement effect leads to enhancement of the electromechanical properties of each crystal structure based on molecular level optimization.

This work was financially supported by the European Research Council through an ERC Starting Grant (ERC-2014-STG-639526, NANOGEN). Y. S. C. is grateful for studentship funding through the Cambridge Commonwealth, European & International Trust. The authors thank Mary Vickers for useful discussions, and Dassault Systèmes BIOVIA for making available BIOVIA Materials Studio under the auspices of EPSRC grant EP/L015552/1. SKK is grateful to CDT Ltd. for funding.

## Conflicts of interest

There are no conflicts to declare.

## Supplementary Material

Supplementary information
