# Supplementary material for: The effect of crystal structure on the electromechanical properties of piezoelectric Nylon-11 nanowires
Source: Chem Commun (Camb). 2018 Jun 1;54(50):6863–6. doi: 10.1039/c8cc02530d (PMC6009497; doi:10.1039/c8cc02530d)
Supplement: Supplementary file 1 [file CC-054-C8CC02530D-s001.pdf]

Electronic Supplementary Material (ESI) for ChemComm.
This journal is © The Royal Society of Chemistry 2018

[Electronic Supplementary Information]

The Effect of Crystal Structure on the Electromechanical Properties of Piezoelectric Nylon-11 Nanowires

Yeon Sik Choi, Sung Kyun Kim, Findlay Williams, Yonatan Calahorra, James A. Elliott, Sohini Kar-Narayan*

Department of Materials Science and Metallurgy, University of Cambridge, 27 Charles Babbage Road, Cambridge CB3 0FS, United Kingdom,

** To whom correspondence should be addressed. E-mail: sk568@cam.ac.uk*

S1. Difference in molecular structure between α and δ′-phase

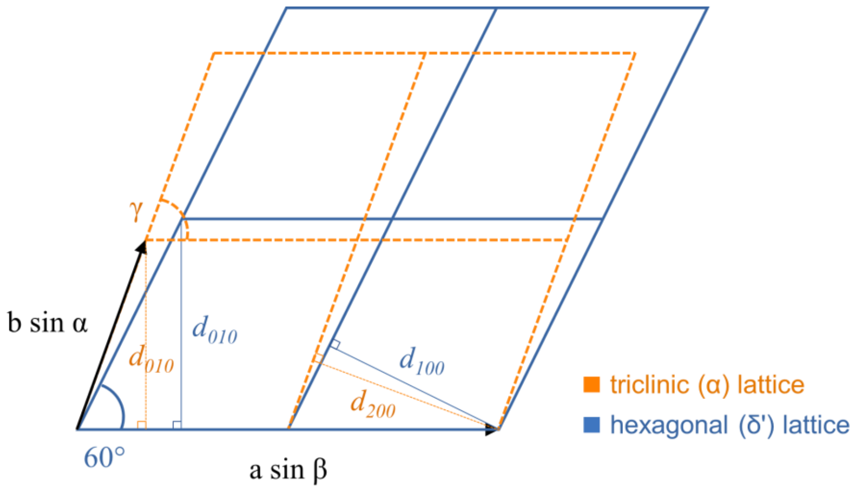

Fig. S1 A comparison between triclinic α-phase lattice (orange) and hexagonal δ′-phase lattice; viewed along the chain axis (proposed by Newman[1]).

At room temperature, Nylon-11 exhibits at least four different crystal structures: the triclinic α and α′, the monoclinic β, and the pseudo-hexagonal δ′ and γ. In this report, we would like to focus on α and δ′-phase Nylon-11 because these two forms display a definite discrepancy in both crystal structure and functional properties.

The α-phase is the most thermodynamically stable configuration of Nylon-11. Initially proposed by Slichter[2] (and then modified by Newman[1]), the α-phase comprises a triclinic unit cell with antiparallel chains (early literature proposes parallel configurations). The hydrogen-bonding between chains forms sheets which progressively shift in the *c* direction (along the chain axis). Unlike other Nylon-11 phases, the α-phase maintains its symmetry and structure up to its melting point (188°C), and is a polar crystal.[3]

The δ′-phase has a pseudo-hexagonal structure. Fig. S1. depicts the differences in lattice structure between the α and δ′-phases, as proposed by Newman[1]. The δ′-phase is characterised by a random distribution of H-bonds along the chain axis, which allows for alignment of dipole moments by external electric field. This phase is metastable, although it remains intact even when left for extended periods of time.[4] It is not yet clear exactly how the H-bond arrays are arranged in the δ′-phases.

S2. Experimental Details

Methods

Materials The Nylon-11 solution was prepared by mixing Nylon-11 pellets (Sigma-Aldrich, M_w = 201.31 g mol^{-1}) and formic acid (Sigma-Aldrich, Reagent grade > 95%) on a hot plate at 90°C. An Anodisc AAO (anodic aluminium oxide, Whatman) 25mm template was used to form the nanowires, with a pore width of 200 nm.

Fabrication of nanowires by conventional template wetting The AAO template was placed on top of the 10 wt% Nylon-11 solution. This assembly was then left at room temperature with no additional gas flow.

Fabrication of α-phase nanowires To make α-phase nanowires, 5 wt% Nylon-11 solution was dropped onto a cleaned petri as described in the conventional template wetting method. However, in this method the AAO template was attached to a square glass slide to limit the exposure of the top surface of the AAO template to the air, thus limiting the rate of formic acid evaporation. A lid was then placed over the petri dish to further reduce exposure to the surrounding air, and the dish was then placed on a hot plate which was pre-heated to 40°C.

Fabrication of δ′-phase nanowires To make δ′-phase nanowires, a bespoke fabrication process[5] was utilised. An AAO template was placed on top of a drop of 17.5wt% Nylon-11 solution in accordance with the conventional template wetting method. No additional protective layers were added, and the solution was not heated. This dish was then placed in a fume cupboard, and a desktop fan was positioned next to the assembly such that air was blown over the surface of the template. The fan was set to blow air at a speed of ~ 3 m s^{-1}, and an anemometer was used to record the flow velocity.

Fabrication of α-phase Nylon-11 films The α-phase Nylon-11 films were produced by drop-casting ~~a~~ 10 wt% solution on the hot plate (40°C).

Preparation of template-freed nanowires This was achieved by first pouring a 40 vol% phosphoric acid solution over the sample and left for 3-4 hours. The resulting nanowire mat was then washed in distilled water and left to air-dry.

Characterization

The field-emission scanning electron microscopy (FE-SEM, FEI Nova Nano SEM) was used to investigate the morphology of the nanowires. The crystal structure of Nylon-11 nanowires with and without the AAO template was observed by an X-ray diffraction (XRD) machine (Bruker D8) using Cu Ka radiation (l = 1.5418 A) with a silicon substrate. QNM and PFM measurements were carried out using Bruker Multimode 8 with Antimony (n) doped Si (tip radius ~ 35 nm, resonance frequency 150 kHz). AC voltages were applied from a lock-in amplifier. To measure the piezoelectric response, one side of the Nylon-11 NW filled AAO template and the α-phase film was coated by sputter (using k550 Emitech).

S3. XRD patterns of α-phase film

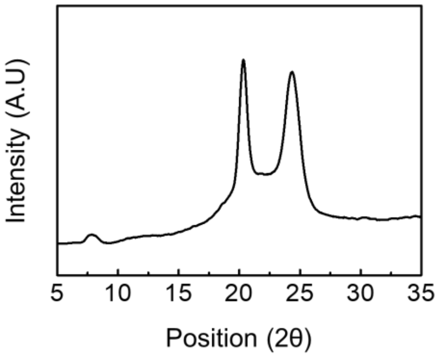

Fig. S3 The XRD pattern of an α-phase Nylon-11film.

The α-phase film produces two distinct peaks at 2θ = 20.02° and 23.01°, corresponding to the (200) and (210/010) planes respectively. There is also a small peak at 7.5°, corresponding to the (001) plane.

S4. Direction of molecular orientation

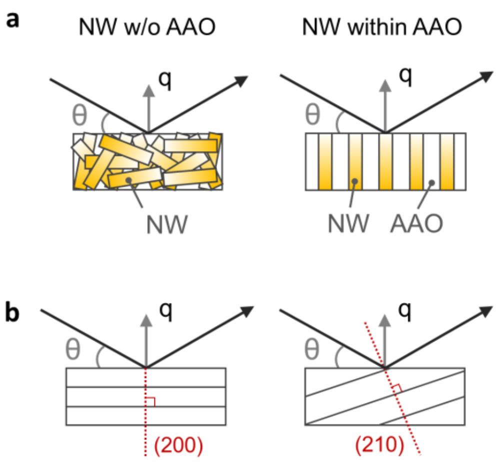

Fig. S4 (a) Schematics for the orientation of nanowire (left) without and (right) within a nanoporous template. Arrows display the direction of x-ray beam (*θ*) with a scattering vector (*q*). (b) The direction of lattice planes and x-ray diffraction.

Nanowires without AAO sample shows all possible diffraction peaks because nanowires are randomly oriented in the sample. In contrast, only family of peaks in the diffraction patterns are observed from the nanowires within AAO. (Fig. S4a)

Fig. S4b depicts the x-ray diffraction with different lattice directions. In the case of (200) plane, a Bragg-Brentano diffractometer produce a diffraction peak at corresponding 2θ angle as Bragg's law is fulfilled. The (210) plane would diffract, however, only background peak is observed because the lattice planes are not aligned with scattering vector (*q*).

S5. Crystallite size calculation

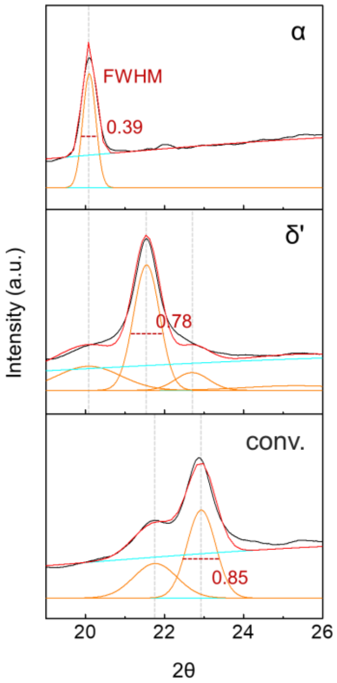

Fig. S5 XRD peak analysis of Nylon-11 nanowires with different crystal structures.

Based on the information of x-ray diffraction peaks, the size of corresponding crystals is calculated by Scherrer equation: $D_p = (0.94 \times \lambda)/\ (\beta \cos\theta)$, where D_p is average crystallite size, β is line broadening in radians, θ is Bragg angle, and λ is X-ray wavelength (= 0.15418 for Cu K-α).

**Phase**	**2θ (°)**	**FWHM**	**Crystal size (nm)**
**α**	20.10	0.39	21.62
**δ'**	21.55	0.78	10.83
	20.09	1.89	4.45
	22.70	1	8.47
**Conv.**	22.93	0.85	9.96
	21.76	1.28	6.56

S6. QNM calibration

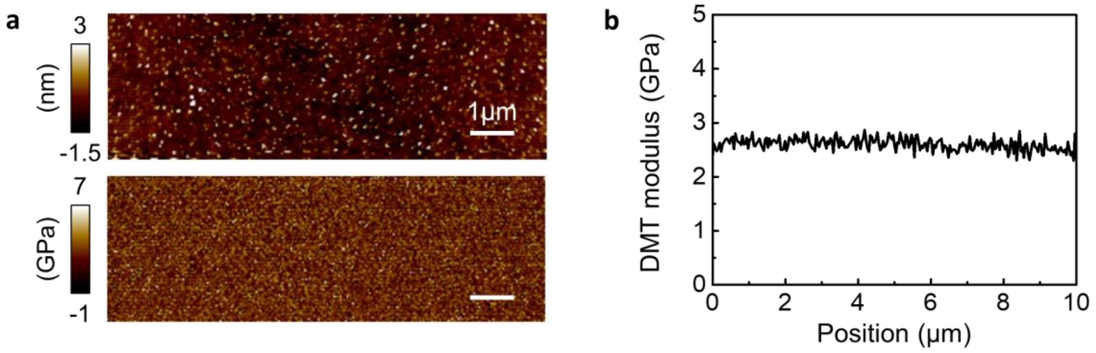

Fig. S6 QNM characterization of the calibration sample (PS). (a) Topology (above) and DMT modulus (below) mapping of the surface of PS. (b) DMT modulus of PS as a function of position.

QNM calibration was performed according to the manufacturer's (Bruker) instructions. First of all, the tip calibration (including deflection sensitivity measurement and thermal tune) was conducted using standard sapphire samples. Then, to calibrate the measuring condition, the DMT modulus of a Polystyrene (PS) film (Bruker) was recorded. Since the DMT value of a standard PS film is already known (2 ~ 3 GPa), we tuned parameters such as peak force amplitude and frequency to reach the specified DMT value. Fig. S6 shows QNM mapping of a standard PS sample. A DMT modulus of 2.6 GPa was achieved via calibrated scanning.

S7. QNM data for Nylon-11 nanowires

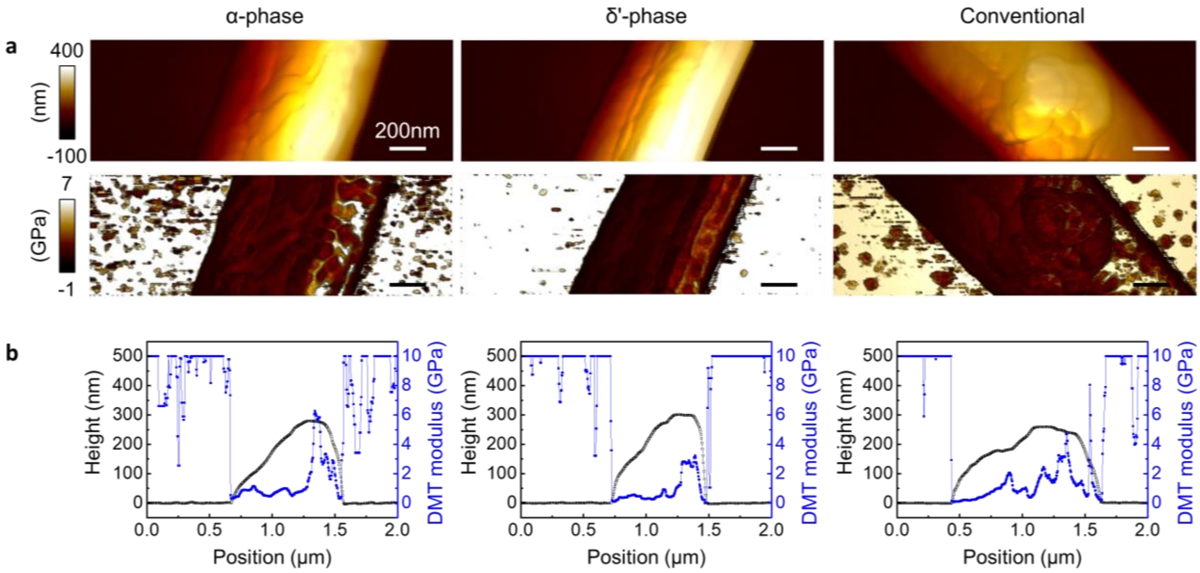

Fig. S7 QNM characteristics of various nanowire phases. (a) Topology (above) and DMT modulus (below) mapping of α-phase, δ′-phase and conventional nanowires. (b) Average DMT modulus along the whole width of each nanowire.

S8. QNM data for Nylon-11 film

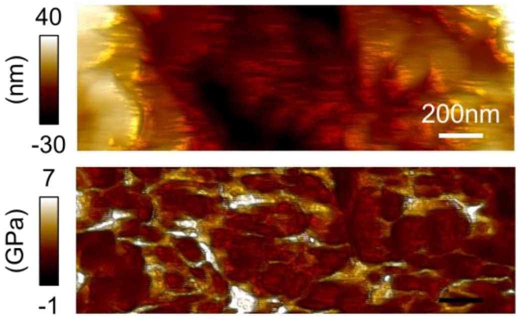

Fig. S8 QNM characteristics of α-phase Nylon-11 film: Height (above) and DMT modulus (below) maps are illustrated.

S9. QNM data for Nylon-11 nanowire and film – height and deformation

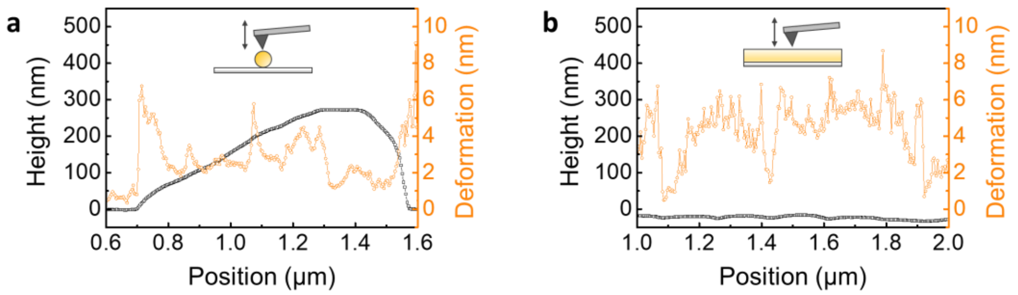

Fig. S9 QNM characteristics of (a) an α-phase Nylon-11 nanowire and (b) an α-phase Nylon-11 film: The plots indicate height (black) and deformation (orange).

S10. PFM for δ′-phase nanowires

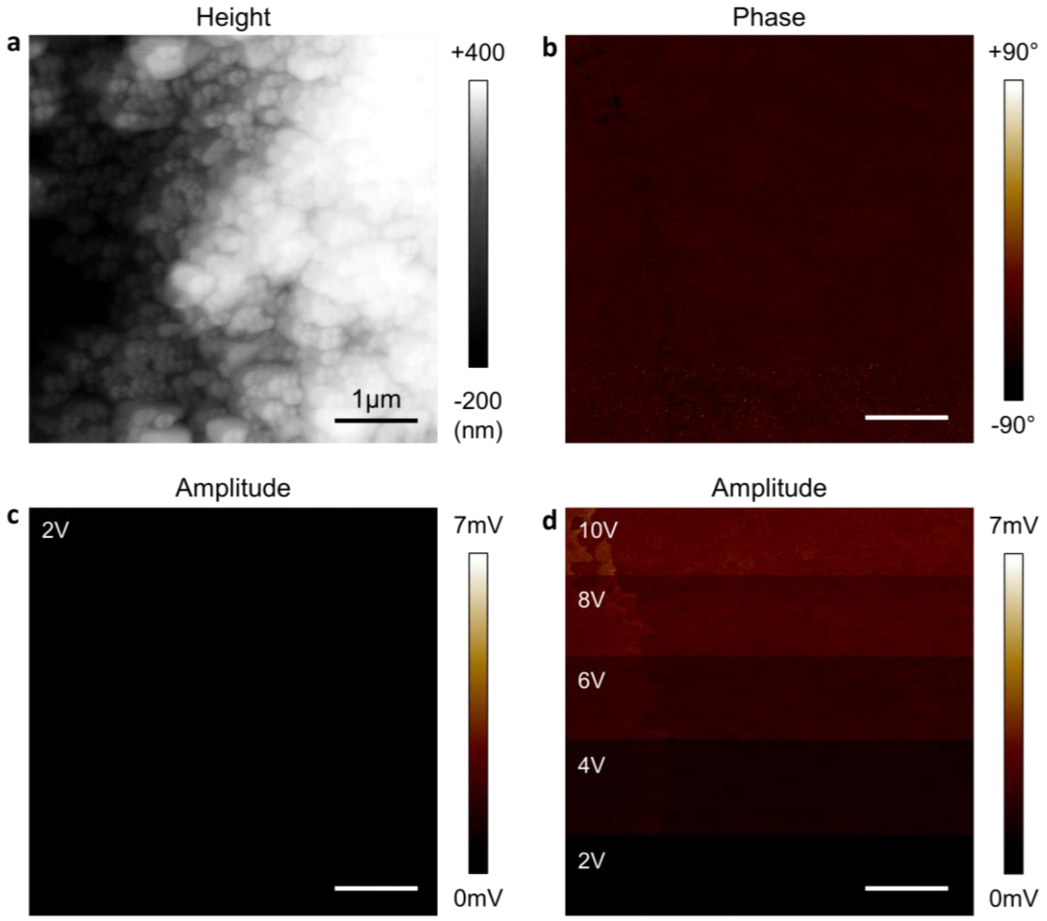

Fig. S10 PFM characteristics of top surface of AAO template filled with δ′-phase nanowires. (a) Height, (b) phase mapping, (c) piezo-response amplitude at AC amplitude of 2V and (d) piezo-response amplitude at various AC amplitude.

S11. PFM calibration

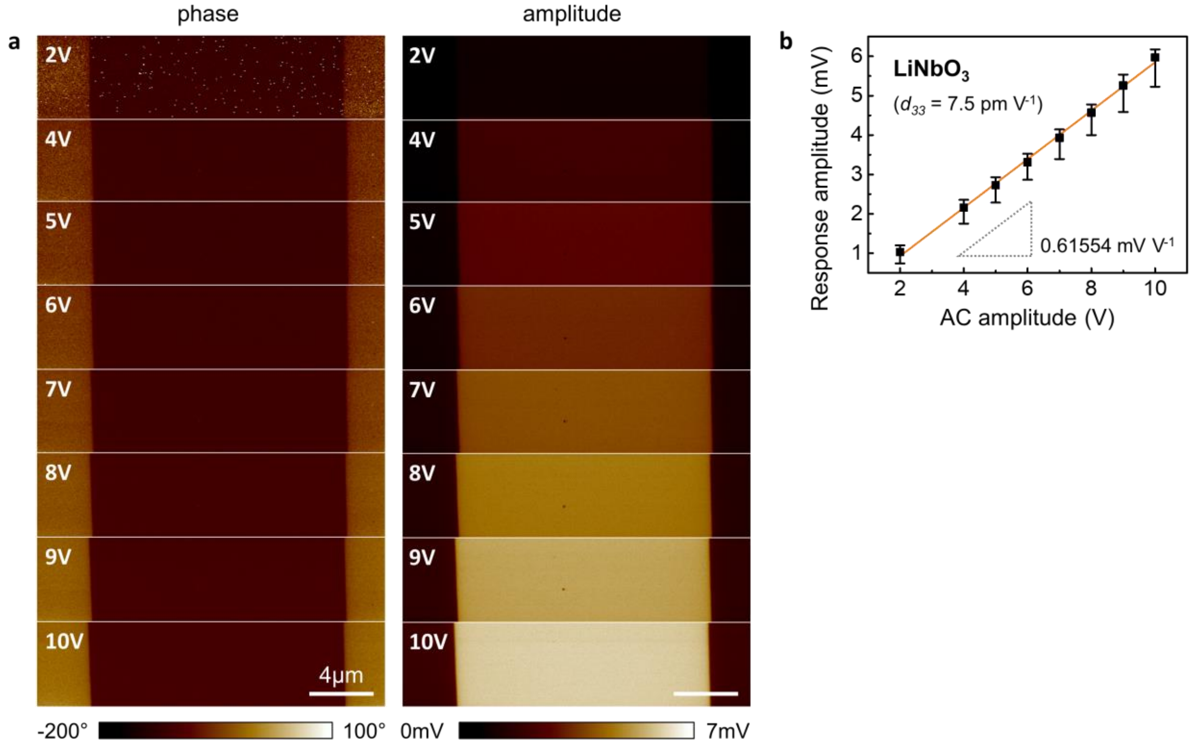

Fig. S11 PFM characteristics of calibration sample ($LiNbO_3$). (a) Phase (left) and amplitude (right) mapping as a function of AC amplitude. (b) Piezoelectric response amplitude of $LiNbO_3$ as a function of AC amplitude.

To calibrate the PFM, a periodically poled lithium niobate ($LiNbO_3$) calibration sample (Bruker) was used. By tuning the phase of lock-in amplifier, we can the maximum the in-phase piezoelectric signals without the out-of-phase component.[6,7] It is noted that the slight increasing trend of PFM response amplitude can be also observed in non-piezoelectric due to the internal voltage coupling of the PFM measuring facility.[6] Therefore, the measurement of surface potential and calibration using non-piezoelectric materials should be preceded.

A linearly increasing trend of vertical PFM response can be achieved by ramping the AC amplitude. To account for the difference between the zero-to-peak applied voltage input and the RMS output of the lock-in amplifier, the PFM response amplitude needed to be corrected by a factor of √2. The slope of the resultant trend (0.61554 mV/V) and piezoelectric coefficient of $LiNbO_3$ (d_{33} = 7.5 pm/V) allows us to calculate a vertical sensitivity of 82 μV/pm.

S12. PFM data for α-phase nanowires

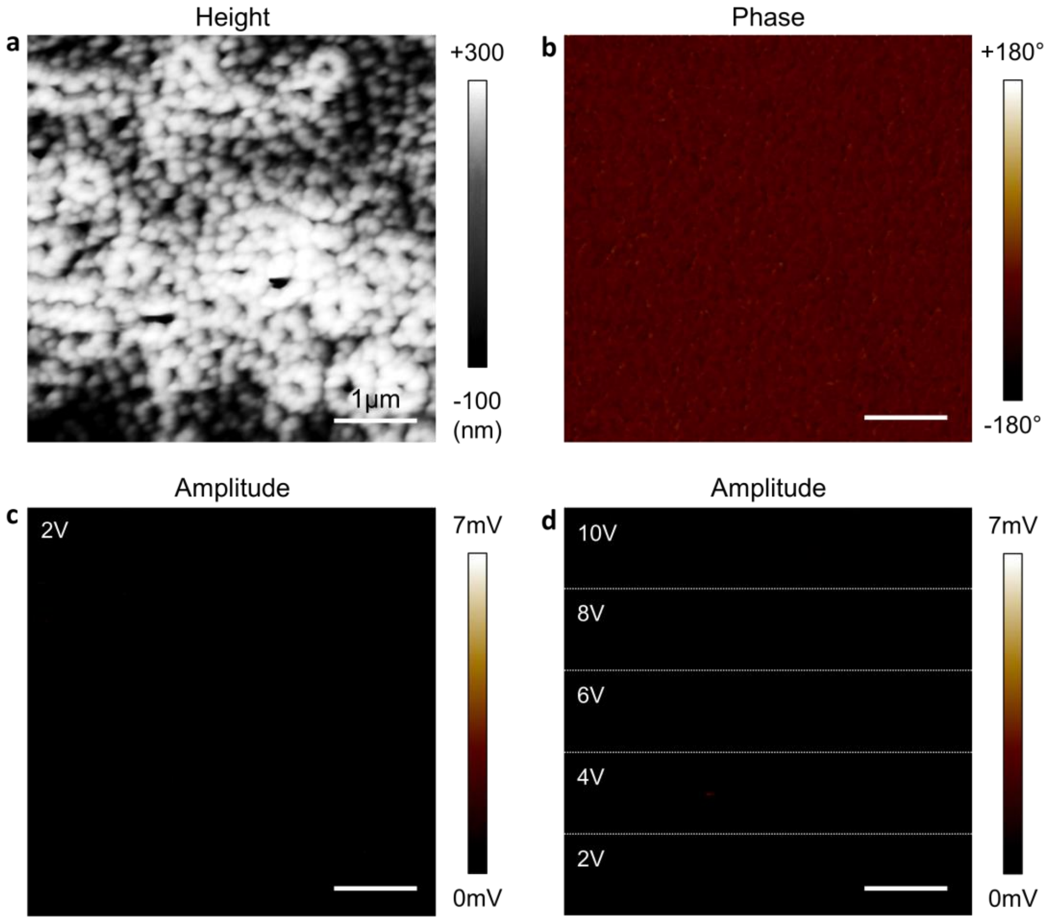

Fig. S12 PFM characteristics of top surface of AAO template filled with α-phase nanowires. (a) Height, (b) phase mapping, (c) piezo-response amplitude at AC amplitude of 2V and (d) piezo-response amplitude at various AC amplitude.

S13. PFM for nanowires by conventional template wetting

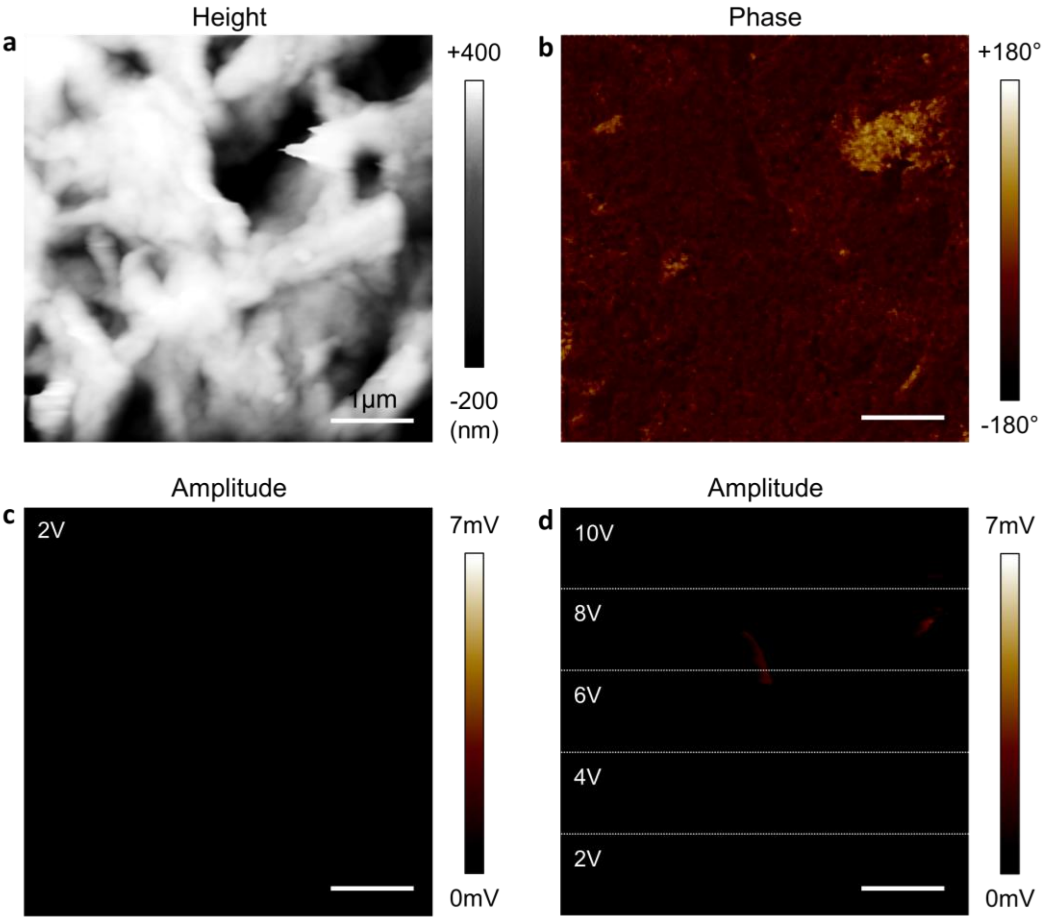

Fig. S13 PFM characteristics of top surface of AAO template filled with nanowires by conventional template wetting. (a) Height, (b) phase mapping, (c) piezo-response amplitude at AC amplitude of 2V and (d) piezo-response amplitude at various AC amplitude.

[Reference]

1. Newman, B. A. *et al.* A high - pressure x - ray study of Nylon 11 A high-pressure x-ray study of Nylon 11. *J. Appl. Phys.* **48,** 4092–4098 (1977).
2. W.P. Slichter. Crystal structures in polyamides made from ω-amino acids. *J. Polym. Sci. Part A Polym. Chem.* **36,** 259–266 (1959).
3. Pepin, J., Miri, V. & Lefebvre, J. M. New Insights into the Brill Transition in Polyamide 11 and Polyamide 6. *Macromolecules* **49,** 564–573 (2016).
4. Kawaguchi, a., Tokimitsu, I., Fujiwara, Y., Tabuchi, M. & Monobe, K. Polymorphism in lamellar single crystals of Nylon 11. *J. Macromol. Sci. Part B Phys.* **20,** 1–20 (1981).
5. Choi, Y. S., Jing, Q., Datta, A., Boughey, C. & Kar-Narayan, S. A triboelectric generator based on self-poled Nylon-11 nanowires fabricated by gas-flow assisted template wetting. *Energy Environ. Sci.* **10,** 2180–2189 (2017).
6. Jungk, T., Hoffmann, A.´ & Soergel, E. Consequences of the background in piezoresponse force microscopy on the imaging of ferroelectric domain structures. *J. Microsc.* **227,** 72–78 (2007).
7. Smith, M., Calahorra, Y., Jing, Q. & Kar-Narayan, S. Direct observation of shear piezoelectricity in poly-l-lactic acid nanowires. *APL Mater.* **5,** (2017).
